# ST-2191, an Anellated Bismorpholino Derivative of Oxy-Fingolimod, Shows Selective S1P_1_ Agonist and Functional Antagonist Potency In Vitro and In Vivo

**DOI:** 10.3390/molecules26175134

**Published:** 2021-08-24

**Authors:** Bisera Stepanovska Tanturovska, Aleksandra Zivkovic, Faik Imeri, Thomas Homann, Burkhard Kleuser, Holger Stark, Andrea Huwiler

**Affiliations:** 1 Institute of Pharmacology, University of Bern, Inselspital INO-F, CH-3010 Bern, Switzerland; bisera.stepanovska@pki.unibe.ch (B.S.T.); faik.imeri@pki.unibe.ch (F.I.); 2 Institute of Pharmaceutical and Medicinal Chemistry, Heinrich Heine University Düsseldorf, Universitaetsstr. 1, D-40225 Duesseldorf, Germany; aleksandra.zivkovic@hhu.de (A.Z.); stark@hhu.de (H.S.); 3 Institute of Nutritional Science, University of Potsdam, Arthur-Scheunert Allee 114–116, D-14558 Nuthetal, Germany; homann@uni-potsdam.de; 4 Institute of Pharmacy, Freie Universität Berlin, Königin-Luise-Str. 2+4, D-14195 Berlin, Germany; burkhard.kleuser@fu-berlin.de

**Keywords:** sphingosine 1-phosphate, S1P_1_ receptor, functional antagonism, lymphopenia, ST-2191, anellated bismorpholino

## Abstract

Sphingosine 1-phosphate (S1P) is an extensively studied signaling molecule that contributes to cell proliferation, survival, migration and other functions through binding to specific S1P receptors. The cycle of S1P_1_ internalization upon S1P binding and recycling to the cell surface when local S1P concentrations are low drives T cell trafficking. S1P_1_ modulators, such as fingolimod, disrupt this recycling by inducing persistent S1P_1_ internalization and receptor degradation, which results in blocked egress of T cells from the secondary lymphoid tissues. The approval of these compounds for the treatment of multiple sclerosis has placed the development of S1PR modulators in the focus of pharmacological research, mostly for autoimmune indications. Here, we report on a novel anellated bismorpholino derivative of oxy-fingolimod, named ST-2191, which exerts selective S1P_1_ agonist and functional antagonist potency. ST-2191 is also effective in reducing the lymphocyte number in mice, and this effect is not dependent on phosphorylation by sphingosine kinase 2 for activity. These data show that ST-2191 is a novel S1P_1_ modulator, but further experiments are needed to analyze the therapeutic impact of ST-2191 in animal models of autoimmune diseases.

## 1. Introduction

Based on the LIPID MAPS classification system, sphingolipids represent one out of the eight categories of lipids [1,2]. They are now increasingly recognized as not only structural components of biological membranes but as bioactive molecules regulating multiple physiological and pathophysiological processes. Sphingosine 1-phosphate (S1P) is an extensively studied example of a signaling sphingolipid that contributes to cell proliferation and survival, migration, differentiation, cellular remodeling and extracellular matrix production [3]. The first step in these cellular responses is the binding of S1P to specific high-affinity cell surface receptors (S1PRs), of which five subtypes have been described, named S1P_1_, S1P_2_, S1P_3_, S1P_4_ and S1P_5_ [4,5]. These receptors belong to the superfamily of G-protein coupled receptors (GPCR). Every cell type and organ so far studied expresses one or several S1PR, which have distinct or overlapping downstream effector molecules; thus, each cell type is influenced in a unique way [6]. Moreover, the concentration of S1P in various body compartments, such as blood or secondary lymphoid organs, varies drastically, which may even trigger opposite reactions by the same cells.

As observed for several other GPCRs, which internalize upon ligand binding and receptor activation [7], S1P_1_ also internalizes upon S1P binding. This is a complex and reversible process, which depends on the concentration gradient of S1P, and is fundamental for T cell trafficking. In environments with high levels of S1P, such as the blood, S1P_1_ is internalized and is not detectable at the surfaces of T cells, which allows homing in secondary lymphoid tissues [8]. In secondary lymphoid tissues, where S1P levels are extremely low, S1P_1_ reappears on the surface and enables sensing of the high gradient of S1P in the lymph [8]. This directs the egress of T cells, i.e., the exit from secondary lymphoid tissues. 

FTY720 (fingolimod) in its phosphorylated form resembles the structure of S1P and was found to activate all S1P receptor subtypes except S1P_2_ [9]. In 2010, fingolimod became the first S1PR modulator that received marketing authorization for the treatment of relapsing–remitting multiple sclerosis [10]. The approval of fingolimod has placed the development of S1PR modulators in the focus of pharmacological research, mostly for autoimmune indications [11]. Recently, the more selective S1PR modulators siponimod, ponesimod and ozanimod were also approved by the European Medicines Agency (EMA) for the treatment of multiple sclerosis, and ozanimod by the FDA for the treatment of ulcerative colitis. The area of S1PR modulators is expanding and molecules with structures related or unrelated to S1P, with unselective or selective affinities, with an agonistic or antagonistic mode of action, are being introduced [5]. Targeting S1P_1_ is of particular interest for the novel modulators, because of the key role of S1P_1_ in T cell trafficking. As opposed to the endogenous ligand S1P, synthetic S1P_1_ modulators, such as fingolimod, siponimod, ozanimod and ponesimod, induce permanent internalization upon binding to and activation of S1P_1_ [12,13,14,15]. Such internalization, which is not followed by recycling to the cell surface, depletes the number of T cells in the blood as they become unresponsive to the S1P gradient [16]. T cells remain in the secondary lymphoid tissues and the result is an immune modulation that is necessary for autoimmune and inflammatory diseases, where T cells play a major pathological role [17]. 

Previously, we developed two morpholino analogues of fingolimod (ST-1893 and ST-1894), which showed a selective S1P_1_ activation profile and sustained S1P_1_ internalization consistent with a functional antagonism. Both compounds induced profound lymphopenia in mice and were efficacious in the experimental autoimmune encephalomyelitis model of multiple sclerosis [18]. Here, we report on two novel compounds, ST-2191, which is an anellated bismorpholino derivative of oxy-fingolimod, and ST-2192, which is a fusion of two oxy-analogues of fingolimod by a *N*,*N*’-dimethylene bridge. ST-2191 potently activated S1P_1_ in the low micromolar range, but it had no effect on any of the other S1PRs. Compared to ST-2191, ST-2192 had only a minor effect on S1P_1_. We further characterized ST-2191 as a functional antagonist of S1P_1_ with in vivo efficacy in reducing the number of lymphocytes in mice. Moreover, and in contrast to FTY720, this substance still induced lymphopenia in Sphk2-deficient mice, thus highlighting its “non-pro-drug” property. Further studies are warranted to confirm the potential of ST-2191 as a selective S1P_1_ modulator in animal models of autoimmune diseases. 

## 2. Results

**Figure 1:**
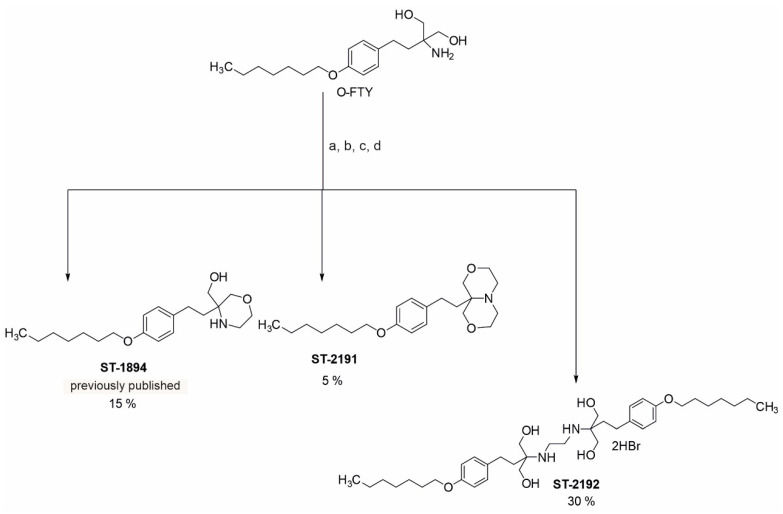
Scheme of the chemical synthesis route for ST-2191 starting from the oxy-analogue of fingolimod (O-FTY). The main product of this reaction is ST-2192 (30%), while ST-1894 (15%) (see also [18]) and ST-2191 (5%) were generated as side products. Synthesis details can be found in the Supplementary File. (a) 1,2-dibromoethane, acetone; (b) crystallization with acetone, DCM/ammonia; (c) extraction with DCM; (d) flash chromatography with DCM/methanol.

ST-2191 (9a-(4-(heptyloxy)phenethyl)hexahydro-1*H*,3*H*-[1,4]oxazino [3,4-*c*][1,4]oxazine) is structurally related to the previously reported S1P_1_ selective modulator ST-1894 (Figure 1) [18]. Both can be synthesized using the oxy-analogue of fingolimod [19] as a precursor (Supplementary File). The same reaction yielded another product, ST-2192, which contains two oxy-analogues of fingolimod fused by a *N*,*N*′-dimethylene bridge.

**Figure 2:**
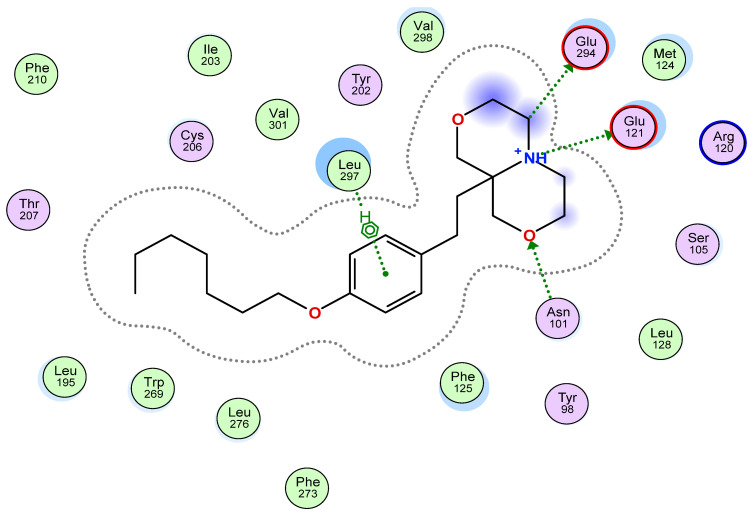
Two-dimensional molecular model of interactions between ST-2191 and amino acid residues of the binding pocket of the S1P_1_ receptor. Molecular modelling experiments were performed as explained in the Materials and Methods section. Green colored circles represent hydrophobic amino acids, while pink circles represent polar amino acids. Red contour is drawn around the acidic and blue around basic amino acids. The blue clouds in the structure of ST-2191 indicate the surface exposed to the solvent, while the blue halo around some amino acids indicates an interaction with the ligand, where a larger halo means a stronger interaction. Hydrogen bonds are represented by green arrows.

A common feature of all S1P receptor modulators so far approved for the treatment of multiple sclerosis or ulcerative colitis is the targeting of S1P_1_. Interaction with S1P_1_ is of primary importance in the development of novel S1PR modulators due to the lymphopenic effects that this interaction may produce [5]. Thus, in a first step, we employed molecular modelling and energy minimization experiments using a homologue model of an activated S1P_1_ receptor (Figure 2, Supplementary Figure S1). The nitrogen atom of the anellated bismorpholino moiety (an oxazino-oxazine) interacted with Glu-121 and Glu294 (H-acceptors), while the oxygen interacted with Asn-101 (H-donor). The phenyl moiety interacted with the hydrophobic residue Leu-297 (π-H). Other amino acids that interact with atoms of ST-2191 include: Val-298, Phe-125, Leu-276, Leu-195, Met-124, Trp-269, Cys-206, Ser-105 and others. This, together with the data from the energy minimization of the whole system (protein S1P_1_ and ST-2191) and the energy calculations of the ligand in the active site performed on it, with the corresponding binding affinity score and binding enthalpy (Supplementary Table S1), indicated a good docking of ST-2191 and predicted an interaction with S1P_1_, which needed to be confirmed in a cellular system. 

**Figure 3:**
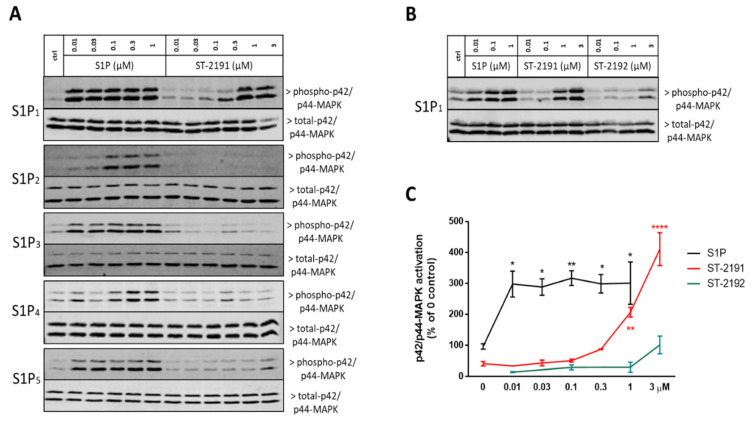
S1P receptor activation profile of ST-2191. CHO cells overexpressing the S1P_1_, S1P_2_, S1P_3_, S1P_4_ or S1P_5_ were stimulated for 10 min with vehicle (DMSO, ctrl), S1P (**A**,**B**), ST-2191 (**A**,**B**) or ST-2192 (**B**) in serum-free medium and were further processed for Western blot detection of phospho-p42/p44-MAPK and total p42/p44, as explained in the Materials and Methods section. Blots show one representative out of three independent experiments. (**C**) Bands of phospho-p42/p44-MAPK were quantified by using ImageJ software. Results are presented as means ± S.E.M. (*n* = 3). * *p* < 0.05, ** *p* < 0.01, **** *p* < 0.0001 compared to DMSO-treated control. A non-linear regression analysis was used to calculate the half-maximal effective concentration (EC_50_) of ST-2191 for the S1P_1_ (R^2^ = 0.96) as 1.99 μM.

To characterize the S1P receptor activation profile of ST-2191, we used CHO-K1 cells, which stably overexpress the different S1P receptor subtypes, i.e., S1P_1_, S1P_2_, S1P_3_, S1P_4_ and S1P_5_. These cells were stimulated with increasing concentrations of the compounds, or with S1P as a positive control. Activation of the receptors was detected by increased phosphorylation of p42/p44-MAPK, which is a well reported early read-out of S1P receptor activation [20,21]. In all S1PR-overexpressing cells, S1P dose-dependently activated the receptors, with effects already seen in the lower nM range, thus confirming previous studies of S1PR characterization [22,23]. ST-2191, up to 3 μM, only activated the S1P_1_, but failed to activate the other receptor subtypes, suggesting that the compound is a selective S1P_1_ agonist (Figure 3A). ST-2192 only slightly activated S1P_1_ at the highest concentration of 3 μM (28% of the ST-2191 effect at 3 μM) and was thus discontinued from further studies (Figure 3B). The half-maximal effective concentration (EC_50_) of ST-2191 for the activation of p42/p44-MAPK in CHO-S1P_1_ was 1.99 μM (Figure 3C).

**Figure 4:**
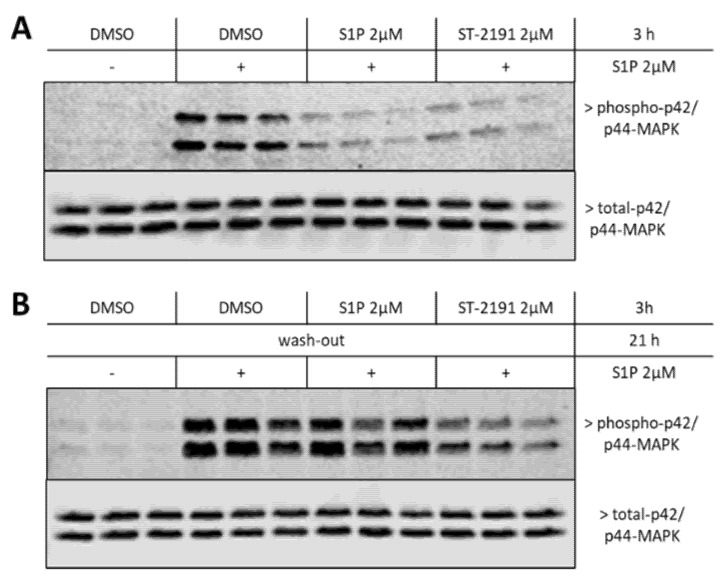
Prolonged effect of ST-2191 on S1P_1_ signaling. Confluent and starved CHO-S1P_1_ cells were stimulated with vehicle (DMSO, ₋), S1P or ST-2191 in a serum-free medium for 3 h (**A**) or 3 h with a wash-out period of 21 h (**B**) and were further stimulated with a new pulse of S1P for 10 min. Cells were lysed and processed for Western blot detection of phospho- and total-p42/p44-MAPK, as explained in the Materials and Methods section.

It was important to analyse whether ST-2191 behaves similarly to fingolimod in inducing sustained internalization of S1P_1_ after the initial step of agonistic activation. Incubation of CHO-S1P_1_ cells for 3 h with either S1P or ST-2191, prior to stimulation with S1P for 10 min, downregulated the phosphorylation of p42/p44-MAPK (Figure 4A) consistent with internalized receptor. When a wash-out step was included after 3 h, and cells were allowed to recover for further 21 h, cells became again responsive to short-term S1P stimulation only in the case of S1P pretreatment/recovery, but not in the case of ST-2191 pretreatment/recovery setup (Figure 4B). This suggests that prolonged ST-2191 treatment, even at a half-maximal effective concentration, desensitizes S1P-induced p42/p44-MAPK activation, possibly through the downregulation of the S1P_1_ receptor’s surface expression. 

**Figure 5:**
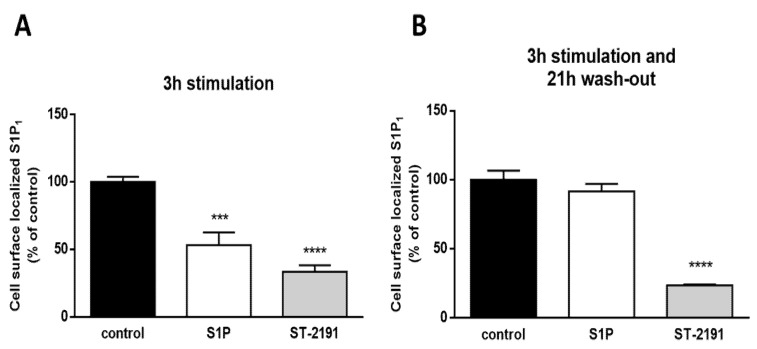
Prolonged effect of ST-2191 on S1P_1_ internalization. Confluent and starved CHO-S1P_1_ cells in 24-well plates were stimulated with DMSO (control), S1P or ST-2191 in a serum-free medium for 3 h (**A**) or 3 h with a wash-out period of 21 h (**B**). After fixation, S1P_1_-ELISA was performed as explained in the Materials and Methods section. Results are expressed as % of control and are depicted as means ± S.D. (*n* = 3; *** *p* < 0.001, **** *p* < 0.0001).

To test this hypothesis, we performed an in situ ELISA on fixed and non-permeabilized CHO-S1P_1_ cells where the S1P_1_ receptor was Myc-tagged on the extracellular domain. ST-2191 reduced the cell surface-expressed S1P_1_ after either 3 h treatment (Figure 5A) or 3 h treatment plus subsequent wash-out/recovery for 21 h (Figure 5B). Moreover, 3 h S1P stimulation decreased cell surface S1P_1_; however, after the wash-out/recovery period, S1P_1_ reappeared on the cell surface. This confirms the previously reported receptor dynamics in the presence and absence of S1P and emphasizes the different internalization patterns triggered by ST-2191. 

**Figure 6:**
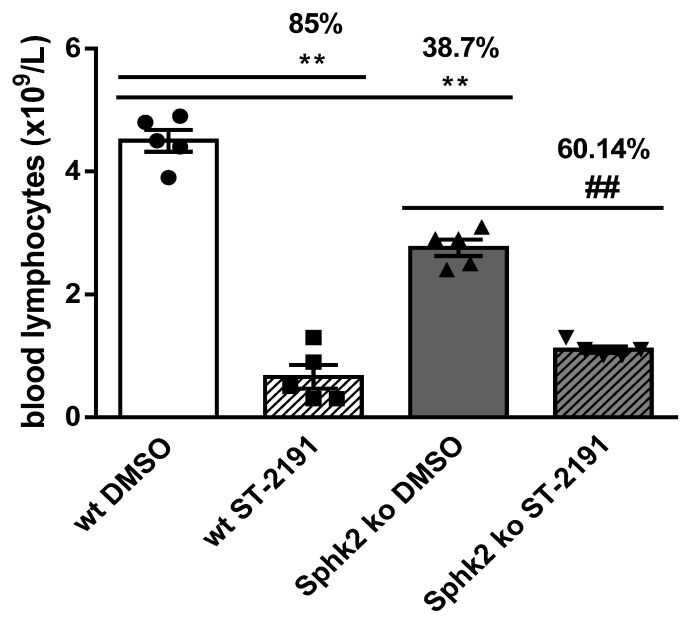
ST-2191-induced decrease in lymphocyte numbers in mice. Wildtype mice (wt) or mice deficient in Sphk2 (Sphk2 ko) were injected in the peritoneum with a single dose of 1 mg/kg ST-2191 (in DMSO/PBS) or vehicle (DMSO in PBS), and after 24 h, blood was collected and lymphocyte number was measured as explained in the Materials and Methods section. Results are presented as means ± S.E.M. (*n* = 5 in each group) and significance was calculated by Mann–Whitney U test (** *p* < 0.01 compared to vehicle-treated wt mice; ^##^ *p* < 0.01 compared to vehicle-treated Sphk2 ko mice).

Based on these data, ST-2191 possesses the structural characteristics necessary for rapid S1P_1_ activation and a subsequent sustained S1P_1_ internalization in vitro. To translate these findings in vivo, we treated mice with a single dose injection of 1 mg/kg ST-2191 and measured the number of lymphocytes in blood after 24 h. The number of lymphocytes was significantly decreased in the ST-2191-treated group compared to the vehicle-treated group (Figure 6), which can be explained by the sustained S1P_1_ internalization property of ST-2191. We suggest that ST-2191 decreases S1P_1_ on the surfaces of T cells and thereby prevents their egress into the blood, leading to lymphopenia. Notably, such a lymphopenic effect is critical for the immunomodulatory action of fingolimod, siponimod and ponesimod in multiple sclerosis, and of ozanimod in ulcerative colitis. 

Several S1PR modulators, such as fingolimod, amiselimod and mocravimod, are prodrugs that exert their immunomodulatory role only after Sphk2-catalyzed phosphorylation [12,24,25]. Therefore, we tested the effect of ST-2191 on lymphocyte number in mice with global Sphk2 knockout and found that the lymphopenic effect was still present, although reduced, in the absence of Sphk2 (Figure 6). This suggests that ST-2191 does not depend on Sphk2 for its immunomodulatory activity, but it does not rule out whether other active metabolites can be formed in vivo. Notably, the dual S1P_1+5_ modulator ozanimod, which is currently in clinical trials for multiple sclerosis, inflammatory bowel disease and COVID-19, is metabolized to two active structures, which produce most of the clinical effects of the drug [26]. 

## 3. Discussion

In this study, using oxy-fingolimod as a precursor, we synthesized ST-2191, a novel compound with an interesting new anellated bismorpholino moiety. This compound showed a selective S1P_1_ agonistic and functional antagonistic activation profile and reduced the lymphocyte number in mice, demonstrating an immunomodulatory effect that is desired for the treatment of various autoimmune and inflammatory diseases [5]. 

ST-2191 can be synthesized in an analogous route to that of ST-1894, a previously reported S1P_1_ receptor modulator with a morpholino moiety (Figure 1) [18]. Furthermore, ST-2191, which is an anellated bismorpholino derivative by the formal condensation of two morpholine rings, i.e., perhydro[1,4]oxazino[3,4-c][1,4]oxazine derivative of oxy-fingolimod, strongly resembles the oxazolo-oxazolo derivative ST-1071 [20], but contains a larger polar head group. The trigger for agonism on the S1P_1_ receptor is associated with an increase in binding pocket volume and ligands with larger volume can have a more hampered dissociation, which could lead to higher activity [18,27]. However, it seems that there is a limitation to the volume of the ligand that can be accommodated in the binding pocket. Notably, we have seen that ST-2192, which is a fusion of two symmetrical molecules of oxy-fingolimod by the *N*-*N*′-dimethylene bridge, cannot properly fit into the molecular model of S1P_1_ and, at 3 μM, can only slightly activate S1P_1_ (Figure 3B). On the contrary, molecular modelling experiments, using a homologue model of an activated S1P_1_, predicted good binding for ST-2191 (Figure 2) and the binding affinity was estimated in a high nM or low µM concentration range. This was confirmed by the activation of S1P_1_ in CHO-S1P_1_ cells measured by phosphorylation of p42/p44-MAPK (Figure 3C). The EC_50_ value of ST-2191 for S1P_1_ activation was determined as 1.99 µM (Figure 3C).

Contrasting the findings for fingolimod, ST-2191 selectively activates S1P_1_ but no other S1P receptors (Figure 3A). All recently approved S1PR modulators have a more restricted activation profile, where targeting S1P_1_ is still in the focus due to the lymphodepleting effects. Conversely, some of the side effects or beneficial effects of fingolimod may be attributed to the unselective engagement with other S1P receptors. In this regard, cerebrovascular constriction during fingolimod treatment is believed to be a S1P_3_-mediated event [28], while interaction with S1P_5_ has been associated with protection against demyelination and the promotion of remyelination in both in vitro and in vivo animal studies [29]. The S1P_4_-mediated effects of fingolimod are, so far, vague [5], although, recently, anti-allergic and anti-inflammatory effects were demonstrated when S1P_4_ was antagonized [30].

ST-2191 conserved its lymphodepleting effect in the absence of Sphk2, unlike fingolimod, which is a pro-drug and becomes active upon phosphorylation by Sphk2 [31]. It seems obvious that, under situations where Sphk2 is inhibited in patients—for example, when treated with a Sphk2 inhibitor—the use of fingolimod-like pro-drugs is contraindicated. However, such inhibitors are not yet approved but have reached clinical study phases for various indications including cancer. Most advanced is the Sphk2 inhibitor ABC294640 (Opaganib, Yeliva^®^), which has received an orphan drug status for the treatment of cholangiocarcinoma and is presently being tested in a phase 2/3 trial for SARS-CoV-2-induced pneumonia (NCT04467840). Hypothetically, *Sphk2* polymorphisms may also hamper the use of S1PR modulator pro-drugs. Thus far, polymorphisms of *Sphk2* have only been described for its promoter region, which affect the expression of the enzyme [32]. Overall, current S1PR modulators have disparate metabolisms, and while some become active without any modifications, others become active after phosphorylation by Sphk2 (fingolimod, mocravimod, amiselimod [24,25]), or a series of reactions by various enzymes to produce active metabolites such as ozanimod [26]. This highlights the need to dissect the in vivo metabolites that are produced following ST-2191 administration.

Altogether, our data show the synthesis and first characterization of ST-2191 as a selective S1P_1_ modulator with in vivo activity on lymphocyte counts. However, it remains to be shown whether ST-2191 has the potential to become a therapeutic drug, and further experiments are needed to analyze the effectiveness of this substance in animal models of autoimmune diseases.

## 4. Materials and Methods

### 4.1. Chemicals and Chemical Synthesis of ST-2191

All chemicals are listed in the Supplementary File. The chemical synthesis steps of ST-2191 are summarized in Figure 1 and described in detail in the Supplementary File.

### 4.2. Molecular Modelling

Molecular modelling, energy minimization experiments and dynamics simulations were performed as previously explained [33].

### 4.3. Cell Culture

Chinese hamster ovary (CHO)-K1 cells overexpressing human S1P_1_, S1P_2_, S1P_3_, S1P_4_ or S1P_5_ receptor were a kind gift from Dr. Danilo Guerini (Novartis Institutes for Biomedical Research, Basel, Switzerland). CHO-S1P_1_, CHO-S1P_4_ and CHO-S1P_5_ were cultivated in MEM alpha with nucleosides and stable glutamine supplemented with 10% FBS, 10 mM HEPES, 50 µg/mL gentamycin and 0.5 mg/mL G418 for selection. CHO-S1P_2_ and CHO-S1P_3_ were grown in RPMI-1640 with the same supplements as above. Cells were detached from the flasks by 0.25% Trypsin–EDTA and seeded on 60 mm dishes at a concentration of 500,000 cells in 3 mL medium. After achieving confluency, they were placed overnight in a serum-free medium consisting of DMEM, 0.1 mg/mL bovine serum albumin (BSA) and 10 mM HEPES (later abbreviated as serum-free medium).

### 4.4. Cell Stimulation and Western Blot Analysis

After 20 h starvation in serum-free medium, CHO-K1 cells in 60 mm dishes were stimulated with vehicle (DMSO), S1P, ST-2191 or ST-2192 in serum-free medium for 10 min. Thereafter, cells were washed with ice-cold PBS, scraped into lysis buffer and homogenized by sonication, as previously explained [34]. Lysates were centrifuged at 13,000 rpm for 10 min and supernatants were dissolved in 4× Laemmli buffer consisting of 4 mM EDTA, 40 mM Tris pH 7.4, 53% glycerol, 13% SDS, 181 μM DTT and 0.448 mM bromophenol blue to a 1x final concentration. Volumes were adapted in order to equalize the amount of protein after previous quantification according to Bradford. Samples were boiled at 95 °C for 10 min prior to loading in 10% (bis-)acrylamide gels and proteins were separated by SDS-PAGE and transferred to nitrocellulose membrane by semi-dry blotting for 7 min (Trans-Blot Turbo™ by Bio-Rad Laboratories AG, Cressier, Switzerland). Membranes were blocked with 3% (*w*/*v*) low-fat milk powder in PBS for 1 h at room temperature and were incubated overnight at 4^o^C with primary antibody in a blocking buffer consisting of 50 mM Tris-HCl pH 7.4, 200 mM NaCl, 10% (*v*/*v*) horse serum, 3% (*w*/*v*) BSA fraction V and 0.1% (*v*/*v*) Tween20. Phospho-p42/p44-MAPK was from Cell Signaling (No. 4377, dilution 1:1000) and polyclonal antibodies against p42-MAPK and p44-MAPK were in-house generated [35] and used in a dilution of 1:3000. After washing 3 times for 7 min with 0.1% Tween in PBS, IRDye^®^ 800 CW secondary antibody was added, diluted in a blocking buffer at 1:10,000 and was allowed to incubate for 2 h at room temperature. Membranes were washed as before and scanned with an Odyssey machine by LI-COR Biosciences (Bad Homburg, Germany) and the signal intensity was evaluated by ImageJ (National Institutes of Health, Bethesda, MD, USA).

### 4.5. S1P_1_ Internalization ELISA

For the S1P_1_ internalization ELISA, CHO-S1P_1_ cells that stably overexpressed an N-terminally Myc-tagged human S1P_1_ construct were used. The ELISA was performed exactly as described before, but with a change in the concentration of S1P and ST-2191 [18], using Myc-tag primary (Cell Signaling, No. 2276, 1:1000) and anti-mouse HRP-linked secondary antibody (Cell Signaling, No. 7076, 1:5000).

### 4.6. Lymphocyte Count

All animal experiments were approved by the committee of animal experimentation of the Veterinary Department of the Canton of Bern, under approval number BE-50/17. C57BL/6J mice were provided by Janvier Labs (France). Sphk2^tm1geno^ were generated by GenOway S.A. (Lyon, France). C57BL/6J wildtype or Sphk2 knockout mice were injected in the peritoneum with a single dose of 1 mg/kg ST-2191 or DMSO in PBS, and after 24 h, blood was collected from the heart under terminal isoflurane-induced anesthesia in EDTA-K3 coated microvettes (Sarstedt; Sevelen, Switzerland). The number of lymphocytes was measured with a Scil Vet ABC™ Hemocytometer and was expressed per one liter of blood (Scil Animal Care Company GmbH; Viernheim, Germany).

### 4.7. Statistical Analysis

Statistical analysis and graph creation were carried out in GraphPad Prism 6 (La Jolla, CA, USA). Statistical significance was calculated with ordinary one-way ANOVA without matching or unpaired t-test, and data are presented as a mean ± S.D. for n number of independent experiments, unless otherwise stated.

## Data Availability

The authors declare that all data supporting the findings of this study are available within this paper or within the supplementary file, or can be obtained from the corresponding author up on request.

## Supplementary Materials

The following are available online, Figure S1: Ligandscout plot showing interaction of ST-2191 with S1P1; Table S1: Energy calculation with Ligandscout [18,36,37].
