# Supplementary material for: ST-2191, an Anellated Bismorpholino Derivative of Oxy-Fingolimod, Shows Selective S1P1 Agonist and Functional Antagonist Potency In Vitro and In Vivo
Source: Molecules. 2021 Aug 24;26(17):5134. doi: 10.3390/molecules26175134 (PMC8433829; doi:10.3390/molecules26175134)
Supplement: Supplementary file 1 [file molecules-26-05134-s001.zip › molecules-1341552-SI.pdf]

Supplementary File

ST-2191, an anellated bismorpholino derivative of oxy-fingolimod shows selective $S1P_1$ agonist and functional antagonist potency *in vitro* and *in vivo*.

Bisera Stepanovska Tanturovska[1], Aleksandra Zivkovic[2], Faik Imeri[1], Thomas Homann[3], Burkhard Kleuser[3], Holger Stark[2], and Andrea Huwiler[1,*]

[1] Institute of Pharmacology, University of Bern, Inselspital INO-F, CH-3010 Bern, Switzerland;
[2] Institute of Pharmaceutical and Medicinal Chemistry, Heinrich Heine University Düsseldorf, Universitaetsstr. 1, D-40225 Duesseldorf, Germany;
[3] Institute of Nutritional Science, University of Potsdam, Arthur-Scheunert Allee 114–116, D-14558 Nuthetal, Germany.
[4] Institute of Pharmacy, Freie Universität Berlin, Königin-Luise-Str. 2+4, D-14195 Berlin, Germany.
bisera.stepanovska@pki.unibe.ch (B.S.); aleksandra.zivkovic@hhu.de (A.Z.); faik.imeri@pki.unibe.ch (F.I.); homann@uni-potsdam.de (T.H.); burkhard.kleuser@fu-berlin.de (B.K.); stark@hhu.de (H.S.); huwiler@pki.unibe.ch (A.H.)
* Correspondence: huwiler@pki.unibe.ch; Tel.: +41'31'632'32'14

Chemicals and reagents:

Roti®-Quant, Rotiphorese®, DMEM with 4.5 g/L D-glucose and RPMI 1640 medium were bought from Carl Roth GmbH (Karlsruhe, Germany). cOmplete™ protease inhibitor cocktail, fatty acid- and globulin-free bovine serum albumin (BSA), BSA fraction V, dimethyl sulfoxide (DMSO), gentamycin and horse serum, were from Sigma-Aldrich Chemie GmbH (Buchs, Switzerland). MEM alpha was from Thermo Scientific (Waltham, USA); TMB ELISA substrate (fast kinetic rate) from Abcam (Cambridge, UK). S1P was from Avanti Polar Lipids Inc. (Alabaster, USA). Trypsin-EDTA 0.25% and G418 (Geneticin™) were from Life Technologies Limited (Paisley, UK). Fetal bovine serum (FBS) was purchased from PAA Laboratories (Catalogue No. A15-101, Cölbe, Germany). All other basic chemicals were ordered from Carl Roth GmbH (Karlsruhe, Germany) or Sigma Aldrich Chemie GmbH (Buchs, Switzerland).

Chemical synthesis of ST-2191:

1,0 g of O-FTY (3.2 mmol) in 80 mL of 1,2-dibromoethane were refluxed overnight. The reaction mixture was then evaporated to dryness and the compound ST-2192 was crystalized from acetone (30% yield). Acetone solution was evaporated to dryness and partitioned in-between DCM and ammonia (25 % in water). Water phase was extracted twice more with DCM, dried over $MgSO_4$ and evaporated to dryness. Products ST-2192 and ST-1894 were obtained after flash chromatography using DCM/MeOH (saturated with ammonia). The side product ST-2191 could be obtained by the same chromatographic purification. Mass spectra and NMR (^{1}H and ^{13}C) spectra have been determined exactly as previously described [1].

ST-2191: *9a-(4-(Heptyloxy)phenethyl)hexahydro-1H,3H-[1,4]oxazino[3,4-c][1,4]oxazine Hydrochloride*

^{1}H NMR: δ [ppm] (300 MHz, DMSO-d_6): 7.12 (d,J=8.4 Hz, 2H), 6.86 (d, J=8.4 Hz 2H), 4.01-3.25 (m, 12 H), 2.80-2.55 (m, 4H), 1.46 (m, 2H), 1.50 – 1.32 (m, 10H), 0.90 (t, J=7.0 Hz, 3H); ^{13}C NMR (75 MHz, DMSO-d_6) δ 154.32, 136.43, 129.37, 114.98, 69.58, 68.05, 66.70, 60.15, 46.96, 30.60, 29.84, 28.57, 28.19, 27.61, 27.07, 22.81, 14.13; APCI(+): 361.9 ([M+H$^+$]$^+$); Elemental Analysis: Calculated: C (66,40%), H(9.12%), N(3.52%); Found: C(66,06 %), H (9,26%), N(3.14%).

Molecular Modelling:

Molecular modelling experiments were performed using the Molecular Operating Environment (MOE), 2020.09; Chemical Computing Group ULC, 1010 Sherbrooke St. West, Suite #910, Montreal, QC, Canada, H3A 2R7, 2020.

Molecular Dynamics calculation was performend with Yasara (Version 21.4.22) [2]. The energy calculation were carried out with Ligandscout (V.4.4.) [3].

Name	Complex Energy	Binding Site Energy	Binding Affinity Score	Active Conf. Energy	Interaction Energy	Relaxed Conf. Energy	MMFF94 Binding Enthalpy
ST-2191	1194,4	1115,86	-30,38	112,437	-33,894	104,731	-26,188

Suppl. Table S1. Energy calculation with Ligandscout. Indicated as kcal/mol.

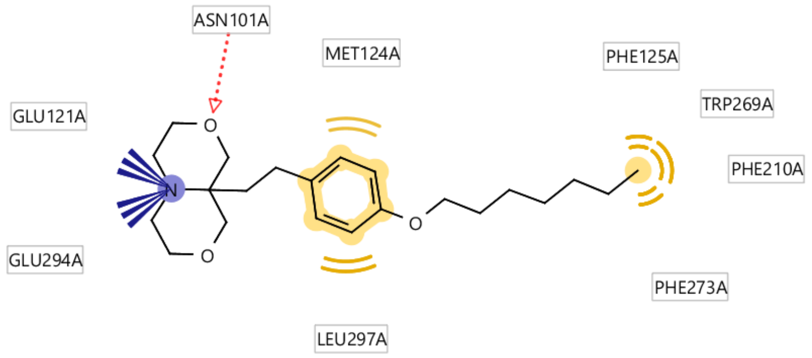

Suppl. Figure S1. Ligandscout plot showing the interaction of ST-2191 with key amio acid residues in $S1P_1$.

References:

1. Stepanovska, B., et al., *Morpholino Analogues of Fingolimod as Novel and Selective S1P1 Ligands with In Vivo Efficacy in a Mouse Model of Experimental Antigen-Induced Encephalomyelitis.* International journal of molecular sciences, 2020. **21**(18): p. 6463.
2. Krieger, E., et al., *Improving physical realism, stereochemistry, and side-chain accuracy in homology modeling: Four approaches that performed well in CASP8.* Proteins: Structure, Function, and Bioinformatics, 2009. **77**(S9): p. 114-122.
3. Wolber, G. and T. Langer, *LigandScout: 3-D pharmacophores derived from protein-bound ligands and their use as virtual screening filters.* Journal of chemical information and modeling, 2005. **45**(1): p. 160-169.
